# Public health policies and scientific evidence

**DOI:** 10.1590/S1679-45082017ED4314

**Published:** 2017

**Affiliations:** 1 Faculdade de Medicina, Universidade de São Paulo. SP Brazil; Programa Diretrizes da Associação Médica Brasileira, São Paulo, SP, Brazil.

Patients are the same all over the world. Evidence is the same in the entire world. However, care policies are not the same. This is impossible to understand and unacceptable, since there is no different evidence for public and private care; it is not a matter of being capitalist or socialist, of presence or distribution of money; it is the fight against consumerist, untruthful and voracious capitalism, encouraging licit “robbery”; it is an issue of equity and ethics; of assured constitutional rights; in which doing something means always doing it correctly and for everyone; where there is no freedom and no prohibition at the same time; where everything starts at standardization; where there is no room for futility; where excesses are banned; where not only is there orientation as to what to do, but also as to what not to do; where the patient is not the means, but the end; where variation by VALUE is combated; and where the concept of VALUE considers costs, as well as benefits and damage.^(^ ^1^ ^)^

Nevertheless, how is it possible to not use evidence in health policies? Since evidence defines what is necessary and what is superfluous; provides options of equivalent benefits and forbidden practices; informs about the estimated amount of benefit and damage, and regarding the adoption of new practices with no benefit; defines the concept of value and the use of money with discrimination; leads the market towards its function and the media towards appropriate criticism; gives orientation as to priorities and strategies; reduces variations in practice and conflicts; and balances interests.^(^ ^2^ ^)^

What has been done? How has the “quasi-evidence” been considered in the definition of access to care? Well, the health authorities are limited in deciding “what and how much is paid (or not paid)”, by means of their “technology” assessment centers, using expert ghost consultants, before, during, and after issuing their opinions based on “God only knows” what - but always aligned with their own interests.

What could and should be done initially with the evidence available?

It is considered that, through the same health authorities, four different and complementary offices would be created with a core purpose of defining what is right or wrong to be offered to all patients, contributing to establishing the key strategies and priorities of the national policy: (1) managerial office in evidence-based medicine; (2) evidence-generation offices; (3) evidence-implementation offices; and (4) conflict-arbitration offices.

Further, there are some requirements for these offices, so that they may survive, influence, disseminate, and guarantee quality care at national level:

– There is a need for infrastructure and human resources (qualitative and quantitative), which can assure the appropriate use of scientific information in care, by means of performance of those involved with their results (benefits and safety) and with structuring the service, conformed to the image of the evidence.
– It is necessary to trust evidence, determined by its strength and consistency, which by producing a lower level of uncertainty, allows one to believe and be secure in the practice recommendations and syntheses.
– External credibility (not commercial marketing) of the service is a factor that should also be present, since patients should be satisfied regarding quality of care and results obtained.
– Healthcare professionals should be homogeneously informed, trained, and skilled to make decisions in each specific situation; moreover, by means of experience accumulated they can appropriately compare practice and evidence. With adequate preparation, there is a greater possibility of reproducing the outcomes described in the evidence, in which doing means always doing the right thing.
– Participation of patients in decision making about their care is compulsory; and in the presence of strong evidence, should have their expectations considered and doubts explained, so that the decision is safely made and without regret. In the absence of evidence or in the case of weak evidence, expectations and medical experience should be shared.^(^ ^3^ ^)^
– The various stakeholders must minimally master knowledge about evidence, or this knowledge, once ignored, will lead to skewed language and understanding of the same evidence. Thus, to know how to interpret the strength of the evidence, with its several components, such as size and variation of results (effect), not only leads to an adequate medical comprehension, but also to suitable communication with patients regarding the degree of uncertainty in the decision to be made.
– Lastly, all need to discuss the concept of value, which despite varying according to the location, tends to be distributed in a “normal” manner. In this debate, a few issues should be on the common agenda of the use of evidence, such as: What is the value of health? How to be mortal? Personalized medicine? Quality of life? What is the value of life? Social values? What is benefit? What damage should be tolerated? This can only be reached through education, consciousness and perception of all being aware that we share the same healthcare system and services, and for any attitudes or actions taken, there will be direct or indirect consequences, near or far for all, hindering the consolidation of equity in health in our country.^(^ ^4^ ^,^ ^5^ ^)^

Although many perceive the agenda to implement evidence in health policies as a simplistic use of a “cookbook”, the effects of no adequate use of evidence speak for themselves. The requirements to implement evidence are fundamentally associated with generation, interpretation, communication, and adequate use. However, three more prerequisites are necessary - which may precede evidence or not - so that doubts, priorities and strategies be focused on local needs:^(^ ^6^ ^–^ ^8^ ^)^

– A precise mapping and diagnosis of distribution of the major health problems, which will guide the priorities according to the local severity and prevalence.
– The record and control of the population's health information, which will contribute to diagnosis besides generating or validating evidence implemented or to be implemented.
– Education actions including training, structuring, combating, defusing, audit, measurements, analyses, reassessments, and follow-up.

Is scientific evidence in public health policy possible? Is this question relevant? Have we already done this? What would be the symptoms of this policy? How to identify when this is being done? What are the practical outcomes?

We can declare that in a country whose health policy is based on evidence, there is disinvestment in the unnecessary and investment where there is need. Overdiagnosis, overtreatment, and undertreatment are combated. There are parameters to help in judicialized conflicts. There is control of the activity and of the industrial health market. There is identification of the opportunity for prevention, research, and innovation, since with standardization underway, one can stop to think. There is no imitation; there is personality, enjoying the advantage of the Internet and of global evidence available. There is control of actions, having as target patient well-being and safety, and not the economy of resources at the expense of damage. There is central control of the responsibility of caring for one's patients. There is an environment where the patients really feel cared for. There is participation of all in monitoring the practical use of evidence when delivering care to the population.^(^ ^3^ ^–^ ^8^ ^)^

We do not need to move from this country. We can - here and now - make way for scientific evidence to determine the actions; for professionals to accompany and produce the results; and for the population to give feedback.

## References

[1] Gray M (2017). Value based healthcare. BMJ.

[2] Oxman AD, Lavis JN, Lewin S, Fretheim A (2009). SUPPORT Tools for evidence-informed health Policymaking (STP) 1: What is evidence-informed policymaking?. Health Res Policy Syst.

[3] Harris C, Ko H, Waller C, Sloss B, Williams P (2017). Sustainability in health care by allocating resources effectively (SHARE) 4: exploring opportunities and methods for consumer engagement in resource allocation in a local healthcare setting. BMC Health Serv Res.

[4] Pearson SD, Rawlins MD (2005). Quality, innovation, and value for money: NICE and the British National Health Service. JAMA.

[5] Welch VA, Akl EA, Pottie K, Ansari MT, Briel M, Christensen R (2017). GRADE equity guidelines 3: considering health equity in GRADE guideline development: rating the certainty of synthesized evidence. J Clin Epidemiol.

[6] Harris C, Allen K, Brooke V, Dyer T, Waller C, King R (2017). Sustainability in Health care by Allocating Resources Effectively (SHARE) 6: investigating methods to identify, prioritise, implement and evaluate disinvestment projects in a local healthcare setting. BMC Health Serv Res.

[7] Lavis JN, Wilson MG, Oxman AD, Lewin S, Fretheim A (2009). SUPPORT Tools for evidence-informed health Policymaking (STP) 4: Using research evidence to clarify a problem. Health Res Policy Syst.

[8] Oxman AD, Schünemann HJ, Fretheim A (2006). Improving the use of research evidence in guideline development: 2. Priority setting. Health Res Policy Syst.

